# Psychological Resilience, Stress, and Pain: The Moderating Role of Positive Affect and Well-Being

**DOI:** 10.1093/geroni/igab046.1121

**Published:** 2021-12-17

**Authors:** Calia Morais, Michael Robinson, Roger Fillingim, Emily Bartley

**Affiliations:** 1 University of Florida, University of Florida, Florida, United States; 2 University of Florida, gainesville, Florida, United States; 3 University of Florida, Gainesville, Florida, United States

## Abstract

Chronic low back pain is the leading cause of disability among older adults. The impact of psychological factors, including high levels of stress, are associated with increased risk for pain. Despite the growing evidence suggesting that psychological well-being is associated with better health outcomes, limited research has examined positive psychological factors in the context of pain among older adults. In this secondary data analyses of we examined the association of perceived stress on pain and physical functioning, and the moderating role of positive affect and well-being (PAW) on these relationships. A total of 60 adults over the age of 60 completed completed questionnaires assessing perceived stress (Perceived Stress Scale) and positive affect and well-being (Neuro-QOL PAW). The Back Performance Scale measured back-related physical functioning and movement-evoked pain. We hypothesized that PAW would be inversely associated with pain outcomes and would moderate the relationship between perceived stress and pain. Bivariate correlations assessed the association between study variables, while the interaction of PAW and perceived stress was examined via linear regression. Age (r=.30), income (r=.28), and being married (r=.32) were associated with higher PAW scores, while there was an inverse association with movement-evoked pain (r=-.28). After controlling for demographic covariates, moderation analysis revealed that higher levels of perceived stress were associated with poorer physical functioning, but only among those with lower positive affect and well-being (b=0.14). As seen, examining the influence of positive psychological functioning on pain-related outcomes has important clinical implications that may promote positive pain adaptation in this population.

